# Impaired detection of happy facial expressions in autism

**DOI:** 10.1038/s41598-017-11900-y

**Published:** 2017-10-17

**Authors:** Wataru Sato, Reiko Sawada, Shota Uono, Sayaka Yoshimura, Takanori Kochiyama, Yasutaka Kubota, Morimitsu Sakihama, Motomi Toichi

**Affiliations:** 1 Department of Neurodevelopmental Psychiatry, Habilitation and Rehabilitation, Kyoto University, Kyoto, Japan; 2 Brain Activity Imaging Center, Advanced Telecommunications Research Institute International, Soraku, Japan; 3 Health and Medical Services Center, Shiga University, Hikone, Japan; 4 Rakuwa-kai Otowa Hospital, Kyoto, Japan; 5 Faculty of Human Health Science, Kyoto University, Kyoto, Japan; 6 The Organization for Promoting Neurodevelopmental Disorder Research, Kyoto, Japan

## Abstract

The detection of emotional facial expressions plays an indispensable role in social interaction. Psychological studies have shown that typically developing (TD) individuals more rapidly detect emotional expressions than neutral expressions. However, it remains unclear whether individuals with autistic phenotypes, such as autism spectrum disorder (ASD) and high levels of autistic traits (ATs), are impaired in this ability. We examined this by comparing TD and ASD individuals in Experiment 1 and individuals with low and high ATs in Experiment 2 using the visual search paradigm. Participants detected normal facial expressions of anger and happiness and their anti-expressions within crowds of neutral expressions. In Experiment 1, reaction times were shorter for normal angry expressions than for anti-expressions in both TD and ASD groups. This was also the case for normal happy expressions vs. anti-expressions in the TD group but not in the ASD group. Similarly, in Experiment 2, the detection of normal vs. anti-expressions was faster for angry expressions in both groups and for happy expressions in the low, but not high, ATs group. These results suggest that the detection of happy facial expressions is impaired in individuals with ASD and high ATs, which may contribute to their difficulty in creating and maintaining affiliative social relationships.

## Introduction

The detection of emotional facial expressions is an initial stage in conscious facial expression processing. The appropriate detection of emotional facial expressions in other individuals plays an important role in effectively understanding their emotional states, regulating social behavior, and creating and maintaining social relationships.

Several psychological studies have shown that typically developing (TD) individuals detect emotional facial expressions more rapidly than neutral facial expressions when using the visual search paradigm with photographic stimuli of facial expressions^1–9^. The visual search is an ecologically valid experimental approach that mimics everyday situations in which one must find a target, and photographic facial expressions are realistic stimuli compared with other types of stimuli, such as schematic drawings^10^. For example, Williams *et al*.^3^ conducted an experiment in which participants examined the lined-up photos of facial expressions and responded to the different expressions in them. The results showed that the reaction time (RT) for detecting a sad or happy expression target in a crowd of neutral face distractors was shorter than that for detecting a neutral face among a group of emotional faces. Several studies showed that this rapid detection of emotional facial expressions depends on emotional significance, not visual features, such as curved mouths in happy expressions^5,6^. For example, Sato and Yoshikawa^5^ presented normal emotional facial expressions (anger and happiness) and control stimuli, termed “anti-expressions”^11^, within crowds of neutral expressions. Anti-expressions were created by applying computer-morphing techniques to photographs of emotional facial expressions to implement visual changes equivalent to those in emotional facial expressions from neutral facial expressions; however, the anti-expressions did not clearly show emotions and were most frequently labeled as neutral expressions in free responses^11^. This method allowed the researchers to determine whether rapid detection of emotional facial expressions was attributable to emotional significance or to visual features. The results of the visual search experiment showed that RTs for detecting normal expressions of anger and happiness were shorter than those for detecting anti-expressions. These results indicate that emotional facial expressions are efficiently detected by TD individuals because of their emotional, rather than visual, characteristics.

Autism spectrum disorder (ASD) is a neurodevelopmental disorder primarily characterized by impairments in social interaction^12^. One of the most obvious features of social impairment is the deficient communication involving emotional facial expressions^13^. Several experimental studies have reported that individuals with ASD showed atypical patterns in the recognition of emotional facial expressions^14–73^, although null findings were also reported^74–88^. Atypical patterns were also reported regarding several other types of facial expression processing, such as the perception of form^89^ and motion^90^ of dynamic facial expressions, facial mimicry^91–94^ and autonomic responses to facial expressions^95–98^, and attentional orienting by gaze with emotional facial expressions^85,99^. In addition, some studies reported that individuals with ASD are impaired in their ability to process subliminally presented facial expressions^98,100,101^. Taken together, even though findings are not entirely consistent, the data imply that individuals with ASD are impaired in various types of emotional expression processing, including the early stages of processing.

However, whether the rapid detection of emotional facial expressions is impaired in individuals with ASD remains unknown. Only a few studies have explored this issue, using the visual search paradigm with photographs of facial expressions, as in studies of TD individuals, and they have failed to show clear deficits in the performance of the ASD group^102–104^. For example, Ashwin *et al*.^102^ tested ASD and TD groups in a visual search experiment in which participants detected angry and happy facial expressions within crowds of neutral expressions. The results showed that the RTs for detecting angry expressions were shorter than those for detecting happy expressions in both the ASD and TD groups. However, because these studies only compared the detection of angry vs. happy expressions, it remains unclear whether the group would have differed in their RTs for emotional vs. neutral facial expressions. Given the aforementioned evidence showing impaired emotional expression processing in ASD groups, we hypothesized that the rapid detection of emotional vs. neutral facial expressions might also be impaired in individuals with ASD.

In addition, it remains to be seen whether detection of emotional facial expressions is similarly affected in individuals from the general population who have high autistic traits (ATs). Several previous studies have shown that the autistic phenotypes are continuously distributed as ATs in the general population, where clinically diagnosed ASD represents the upper extreme^105–108^. Several psychometric scales were developed to quantitatively and reliably measure ATs, such as the autism-spectrum quotient (AQ)^105^. Previous research indicated that individuals with high ATs showed more deficiencies in various types of facial expression processing, including emotion recognition in facial expressions^109^, facial mimicry of emotional expressions^110^, and gaze-triggered attentional orienting by emotional expressions^111^ than those with low ATs. However, no study has yet investigated effects of ATs on the detection of emotional facial expressions. Based on the above data showing similarity in impaired facial expression processing among individuals with ASD and high ATs, we hypothesized that the rapid detection of emotional vs. neutral facial expressions would be impaired in individuals with high ATs.

**Figure 1:**
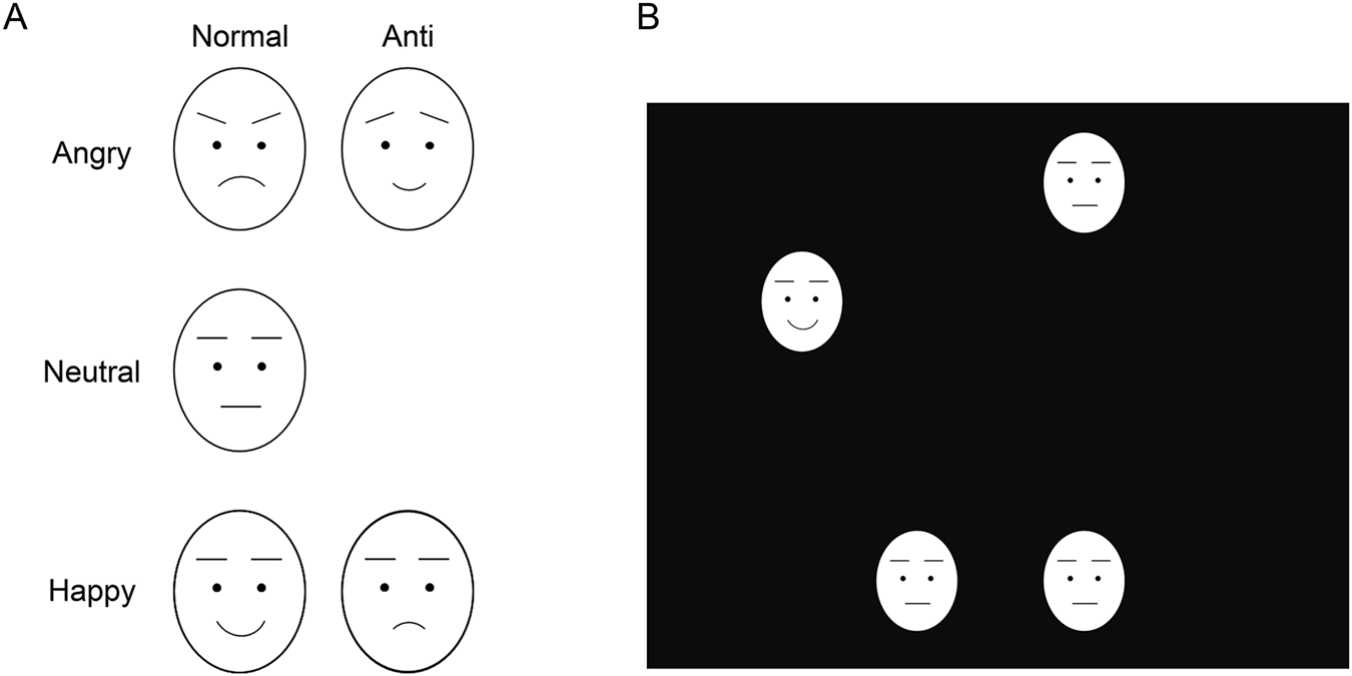
Schematic illustrations of stimuli (**A**) and visual search display (**B**). Actual stimuli were photographs of faces (see Fig. 1 in Sato and Yoshikawa^5^) .

In the present study, we tested these hypotheses by comparing ASD vs. TD individuals in Experiment 1 and non-clinical individuals with high vs. low ATs in Experiment 2 using the visual search paradigm (Fig. 1). We used facial expressions depicting anger and happiness as target stimuli presented within crowds of neutral expressions, as has been done in several previous studies with TD individuals^1–5,7–9^. We also presented anti-expressions as targets, as in previous studies^5,7–9^. Because anti-expressions replicated changes equivalent to those between normal emotional facial expressions and neutral expressions but were less emotional and most frequently labeled as neutral^11^, they allowed us to compare, approximately, emotional vs. neutral facial expressions controlling for the effects of basic visual processing. We predicted that the rapid detection of normal vs. anti-expressions would be evident in the TD group but not in the ASD group in Experiment 1 and in the low ATs group but not in the high ATs group in Experiment 2. To confirm the emotional impact of normal expressions compared with anti-expressions, we additionally required participants to rate the stimuli in terms of subjectively experienced valence and arousal^112^.

## Results

### Experiment 1

In Experiment 1, we compared individuals with high-functioning ASD and age- and sex-matched TD groups for the detection of normal vs. anti-expressions of anger and happiness.

#### RT

**Figure 2:**
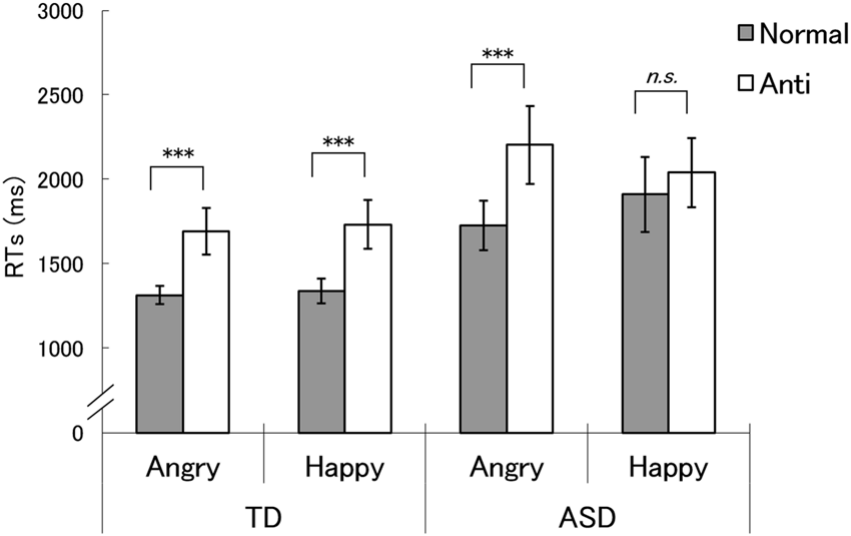
Mean (with *SE*) reaction times (RTs) in the typically developing (TD) and autism spectrum disorder (ASD) groups in Experiment 1. Asterisks indicate the significant simple-simple main effects of stimulus type, indicating shorter RTs for normal expressions than for anti-expressions (***p < 0.001; *n*.*s*.: not significant).

In Fig. 2, the results for RT in Experiment 1 are shown. An analysis of variance (ANOVA) with group (TD, ASD), stimulus type (normal, anti), and emotion (anger, happiness) as factors for log-transformed RTs revealed a significant three-way interaction, *F*(1,32) = 4.24, *p* = 0.048, *η* ^2^_*p*_ = 0.115, indicating that the detection of normal vs. anti-expressions differed depending on the group and the emotion presented. Besides, the main effects of group and stimulus type were significant, *F*(1,32) = 5.81 and 69.89, *p* = 0.022 and 0.000, *η* ^2^_*p*_ = 0.155 and 0.685, respectively, and interactions of group × stimulus type and stimulus type × emotion reached marginal significance, *F*(1,32) = 3.41 and 3.27, *p* = 0.074 and 0.080, *η* ^2^_*p*_ = 0.093 and 0.095, respectively. No other main effects or interactions were significant, *F*(1,32) < 0.26, *p* > 0.1, *η* ^2^_*p*_ < 0.007.

Follow-up analyses were conducted to understand the three-way interaction. First, given our predictions, we investigated the simple-simple main effects of stimulus type. Significant effects, indicating shorter RTs for normal expressions than for anti-expressions, were found for angry expressions in both TD and ASD groups, *F*(1,64) = 27.58 and 30.12, *p* = 0.000 and 0.000, respectively, and for happy expressions in the TD group, *F*(1,64) = 27.62, *p* = 0.000, but not in the ASD group, *F*(1,64) = 2.56, *p* = 0.114. We explored other effects and found that the simple-simple main effects of group were significant only for normal angry, normal happy, and anti-angry expression conditions, indicating shorter RTs for the TD than the ASD groups, *F*(1,128) = 5.40, 9.15, and 5.40, *p* = 0.022, 0.003, and 0.022, respectively. The simple-simple main effects of emotion were significant only for normal and anti-expressions in the ASD group, indicating faster detection of normal angry vs. normal happy expressions and anti-happy vs. anti-angry expressions, *F*(1,64) = 4.59 and 3.99, *p* = 0.036 and 0.049, respectively.

#### Rating

The results of the ratings in Experiment 1 are presented in Table 1. For the valence ratings, an ANOVA with group, stimulus type, and emotion as factors revealed a significant three-way interaction, *F*(1,32) = 11.27, *p* = 0.002, *η* ^2^_*p*_ = 0.260. Besides, the main effects of stimulus type and emotion, and the interactions of group × stimulus type, group × emotion, and stimulus type × emotion were significant, *F*(1,32) = 18.12, 183.41, 8.49, 12.96, and 198.40, *p* = 0.000, 0.000, 0.007, 0.001, and 0.000, *η* ^2^_*p*_ = 0.362, 0.851, 0.210, 0.288, and 0.861, respectively. The remaining main effect of group were not significant, *F*(1,32) = 0.62, *p* = 0.437, *η* ^2^_*p*_

**Table 1:** Mean (with *SE*) subjective ratings of valence and arousal in the typically developing (TD) and autism spectrum disorder (ASD) groups in Experiment 1.

Group	Valence					Arousal				
	Normal		Anti		Neutral	Normal		Anti		Neutral
	Angry	Happy	Angry	Happy		Angry	Happy	Angry	Happy	
TD	2.4 (0.2)	7.9 (0.2)	5.0 (0.3)	5.0 (0.1)	5.0 (0.2)	7.0 (0.4)	7.0 (0.2)	4.9 (0.2)	4.6 (0.3)	3.8 (0.4)
ASD	3.0 (0.3)	6.4 (0.2)	4.9 (0.2)	4.2 (0.2)	5.0 (0.1)	6.2 (0.4)	5.8 (0.2)	5.0 (0.2)	4.5 (0.2)	4.1 (0.3)

= 0.019.

Follow-up simple effect analyses were conducted for the three-way interaction. Significant simple-simple main effects of stimulus type were found for both emotion conditions in both groups, indicating more negative ratings for normal angry expressions and more positive ratings for normal happy expressions than for corresponding anti-expressions in both TD and ASD groups, *F*(1,64) = 74.49, 176.49, 39.73, and 51.49, *p* = 0.000, 0.000, 0.000, and 0.000, respectively. The simple-simple main effects of group were significant only for normal expressions of anger and happiness, indicating more negative ratings for normal angry expressions and more positive ratings for normal happy expressions in the TD than in the ASD group, *F*(1,128) = 4.87 and 24.88, *p* = 0.029 and 0.000, respectively. The simple-simple main effects of emotion were significant for both stimulus-type conditions in both groups, indicating more negative ratings for angry than for happy expressions under normal expression conditions and the reverse under anti-expression conditions in both the in the TD and ASD groups, *F*(1,64) = 289.18, 11.81, 104.48, and 5.53, *p* = 0.000, 0.001, 0.000, and 0.022, respectively.

To compare the valence ratings of normal and anti-expressions of anger and happiness with those of neutral expressions, multiple comparisons with the Bonferroni correction were conducted. The results showed that normal angry expressions were significantly more negative and normal happy expressions were significantly more positive than neutral expressions in the TD and ASD groups, *t*(16) = 17.25, 12.78, 5.85, and 5.57, *p* = 0.000, 0.000, 0.000, and 0.000, *r* = 0.974, 0.976, 0.825, and 0.812, respectively. Anti-angry expressions were not significantly different from neutral expressions in the TD and ASD groups, *t*(16) = 0.00 and 0.14, *p* = 1.000 and 1.000, *r* = 0.000 and 0.035, respectively, whereas anti-happy expressions were rated as significantly more negative than neutral expression in the TD and ASD groups, *t*(16) = 5.37 and 2.93 *p* = 0.000 and 0.040, *r* = 0.802 and 0.591, respectively.

For the arousal ratings, a three-way ANOVA revealed a significant two-way interaction of stimulus type × group, *F*(1,32) = 5.97, *p* = 0.020, *η* ^2^_*p*_ = 0.157. The main effects of group and stimulus type were also significant, *F*(1,32) = 4.7 and 72.15, *p* = 0.038 and 0.000, *η* ^2^_*p*_ = 0.128 and 0.693. There were no other significant main effects or interactions, *F*(1,32) < 2.68, *p* > 0.1, *η* ^2^_*p*_ < 0.078.

Follow-up analyses for the two-way interaction revealed significant simple main effects of stimulus type, indicating higher arousal ratings for normal than for anti-expressions, both in the TD and ASD groups, *F*(1,32) = 59.80 and 18.31, *p* = 0.000 and 0.000, respectively. The simple main effects of group were also significant for normal expressions, indicating higher arousal ratings for normal expressions in the TD than in the ASD group, *F*(1,64) = 10.48, *p* = 0.002.

Bonferroni corrected multiple comparisons contrasting normal and anti-expressions of anger and happiness vs. neutral expressions showed that normal expressions of anger and happiness elicited significantly higher arousal than neutral expressions in the TD and ASD groups, *t*(16) = 7.26, 8.55, 4.74, and 4.53, *p* = 0.000, 0.000, 0.001, and 0.001, *r* = 0.974, 0.976, 0.825, and 0.812, respectively. Anti-angry expressions were rated as eliciting significantly higher arousal than neutral expressions in the TD and ASD groups, *t*(16) = 5.29 and 3.20, *p* = 0.005 and 0.022, *r* = 0.798 and 0.625, respectively. Anti-happy expressions were not significantly different from neutral expressions in the TD and ASD groups, *t*(16) = 2.59 and 1.36, *p* = 0.152 and 0.775, *r* = 0.544 and 0.322, respectively.

### Experiment 2

In Experiment 2, we compared the non-clinical samples of individuals with high vs. low ATs, divided using a median split for the AQ scores, in terms of the detection of normal vs. anti-expressions of anger and happiness.

#### RT

**Figure 3:**
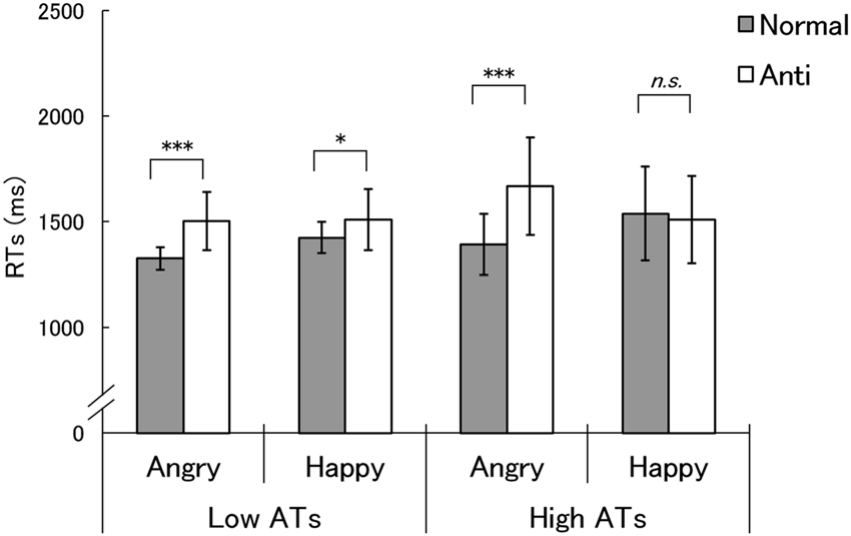
Mean (with *SE*) reaction time (RTs) in the low and high autistic traits (ATs) groups in Experiment 2. Asterisks indicate the significant simple-simple main effects of stimulus type, indicating shorter RTs for normal expressions than for anti-expressions (***p < 0.001; *p < 0.05; *n*.*s*.: not significant).

The results for RT in Experiment 2 are shown in Fig. 3. An ANOVA with group, stimulus type, and emotion as factors for log-transformed RTs showed, as in the results of Experiment 1, a significant three-way interaction between group, stimulus type, and emotion, *F*(1,36) = 4.83, *p* = 0.035, *η* ^2^_*p*_ = 0.118. The main effect of stimulus type and the interaction of stimulus type × emotion were also significant, *F*(1,36) = 47.73 and 17.76, *p* = 0.000 and 0.000, *η* ^2^_*p*_ = 0.543 and 0.330, respectively. No other main effects or interactions were significant, *F*(1,36) < 1.85, *p* > 0.1, *η* ^2^_*p*_ < 0.050.

Follow-up analyses were conducted for the three-way interaction. First, the results revealed significant simple-simple main effects of stimulus type, indicating shorter RTs for normal expressions than for anti-expressions, for angry expressions in both the low and high ATs groups, *F*(1,72) = 18.53 and 39.74, *p* = 0.001 and 0.000, respectively, and for happy expressions in the low ATs group, *F*(1,72) = 4.75, *p* = 0.033, but not in the high ATs group, *F*(1,72) = 0.20, *p* = 0.655. We also explored other effects, and found that there was no significant simple-simple main effect of group, *F*(1,144) < 1.21, *p* > 0.1. The simple-simple main effects of emotion were significant for normal expressions in both the low and high ATs groups, indicating faster detection of angry expressions than happy expressions, *F*(1,72) = 5.39 and 12.42, *p* = 0.024 and 0.001, respectively, and also for anti-expressions in the high ATs group, indicating a faster detection of anti-happy than anti-angry expressions, *F*(1,72) = 12.32, respectively, *p* = 0.001.

#### Rating

The results of the ratings from Experiment 2 are presented in Table 2. For the valence ratings, an ANOVA with group, stimulus type, and emotion as factors revealed a significant two-way interaction of stimulus type × emotion, *F*(1,36) = 89.25, *p* = 0.000, *η* ^2^_*p*_ = 0.713. The main effects of stimulus type and emotion were also significant, *F*(1,36) = 10.19, and 62.32, *p* = 0.003 and 0.000, *η* ^2^_*p*_ = 0.221 and 0.634, respectively. The main effect of group, indicating higher ratings in the low than the high ATs groups, was also significant, *F*(1,36) = 5.91, *p* = 0.020, *η* ^2^_*p*_ = 0.141. There were no other significant interactions, *F*(1,36) < 0.68, *p* > 0.1, *η* ^2^_*p*_

**Table 2:** Mean (with *SE*) subjective ratings of valence and arousal in the low and high autistic traits (ATs) groups in Experiment 2.

Group	Valence					Arousal				
	Normal		Anti		Neutral	Normal		Anti		Neutral
	Angry	Happy	Angry	Happy		Angry	Happy	Angry	Happy	
Low ATs	3.7 (0.3)	6.7 (0.4)	5.1 (0.3)	4.5 (0.2)	5.3 (0.2)	6.3 (0.4)	6.1 (0.3)	4.8 (0.2)	5.1 (0.2)	4.5 (0.3)
High ATs	3.0 (0.3)	6.5 (0.2)	4.6 (0.2)	3.7 (0.2)	5.0 (0.2)	6.1 (0.3)	5.9 (0.2)	4.6 (0.2)	4.5 (0.2)	4.1 (0.2)

< 0.019.

Follow-up analyses were conducted for the stimulus type × emotion interaction and revealed the significant simple main effects of stimulus type for both angry and happy emotions, indicating more negative ratings for normal angry expressions than for anti-angry expressions and more positive ratings for normal than for anti-happy expressions, *F*(1,72) = 150.96 and 8.70, *p* = 0.000 and 0.004, respectively. The simple main effects of emotion were also significant for both normal and anti-expression stimuli, indicating more negative ratings for angry vs. happy expressions among normal expressions and the reverse for anti-expressions, *F*(1,72) = 32.96 and 90.44, *p* = 0.000 and 0.000, respectively.

Bonferroni corrected multiple comparisons contrasting the valence ratings of normal and anti-expressions of anger and happiness vs. those of neutral expressions revealed that normal angry expressions were significantly more negative and normal happiness expressions were significantly more positive than neutral expressions in the low ATs group, *t*(17) = 4.59 and 2.80, *p* = 0.000 and 0.000, *r* = 0.744 and 0.562, respectively, and the high ATs group, *t*(19) = 6.31 and 4.69, *p* = 0.000 and 0.001, *r* = 0.823 and 0.732, respectively. Anti-angry expressions were not significantly different from neutral expressions both in the low and high ATs groups, *t*(17) = 0.38, *p* = 1.000, *r* = 0.092 and *t*(19) = 1.82, *p* = 0.336, *r* = 0.385, respectively. Anti-happy expressions were rated more significantly negative than neutral expression in the low and high ATs groups, *t*(17) = 4.24, *p* = 0.002, *r* = 0.717 and *t*(19) = 4.77, *p* = 0.001, *r* = 0.738, respectively.

For the arousal ratings, a three-way ANOVA revealed the significant main effect of stimulus type, indicating higher arousal ratings for normal than for anti- expressions, *F*(1,36) = 57.41, *p* = 0.000, *η* ^2^_*p*_ = 0.615, and marginally significant main effect of group, *F*(1,36) = 3.47, *p* = 0.071, *η* ^2^_*p*_ = 0.088. No other main effects or interactions were significant, *F*(1,36) < 0.80, *p* > 0.1, *η* ^2^_*p*_ < 0.023.

Bonferroni corrected multiple comparisons for the arousal ratings revealed that normal angry and happy expressions elicited significantly higher arousal than neutral expressions in the low ATs group, *t*(17) = 3.74 and 3.50, *p* = 0.007 and 0.012, *r* = 0.672 and 0.647, respectively, and the high ATs group, *t*(19) = 4.55 and 7.48, *p* = 0.001 and 0.000, *r* = 0.722 and 0.864, respectively. Both anti-expressions of anger and happiness were not significantly different from neutral expressions in the low ATs group, *t*(17) = 0.81 and 1.93, *p* = 1.000 and 0.280, *r* = 0.193 and 0.424, respectively, and the high ATs group, *t*(19) = 1.24 and 1.61, *p* = 0.920 and 0.500, *r* = 0.274 and 0.346, respectively.

## Discussion

Our results in the TD groups in Experiments 1 and 2 consistently showed that normal facial expressions of anger and happiness were detected faster than the corresponding anti-expressions. The subjective emotional rating data confirmed that normal expressions of anger and happiness were more emotionally significant in terms of valence and arousal dimensions compared with the corresponding anti-expressions and neutral expressions. Although anti-expressions were not completely equal to neutral expressions, their responses were restricted to either valence or arousal dimensions and did not satisfy normal emotional elicitation requiring both dimensions^113^. These results are consistent with several previous studies^5,7–9^ and indicate the existence of psychological mechanisms for the efficient detection of emotional facial expressions in TD individuals. The faster detection of normal angry vs. normal happy expressions was evident in Experiment 2 but not in Experiment 1. These inconsistent results are understandable, given previous findings reporting positive^5,8,9^ and null^7^ findings using a similar paradigm. Emotional differences may be less evident and susceptible to influence by other factors, such as individual differences^8,9^, than the difference between normal vs. anti-expressions.

More importantly, our results showed that normal happy expressions were not detected faster than anti-happy expressions in the ASD group. Normal angry expressions were detected faster than anti-angry expressions and than normal happy expressions in the ASD group. The faster detection of angry than happy expressions is consistent with the results of previous studies using the visual search paradigm with angry and happy facial expressions in ASD groups^102–104^. However, these studies did not compare detection performance for emotional and neutral facial expressions. The finding of impaired processing of happy facial expressions in the ASD group supports previous findings that show abnormal patterns in emotion recognition^64,67,71^, motion perception^90^, and facial^92–94^ and autonomic^97^ responses during the processing of happy facial expressions in ASD individuals. However, none of these studies tested the detection of emotional facial expressions. Although impairment in happy expression processing was not consistently shown in previous studies, discrepant results may be explained by methodological differences, such as the ceiling effect for happy expressions in the standard emotion recognition tasks^56,69^. To the best of our knowledge, this is the first study reporting the impaired rapid detection of happy facial expressions in individuals with ASD.

Our results in Experiment 2, as in the results of the ASD group in Experiment 1, showed that normal happy expressions were not detected faster than anti-happy expressions in the high ATs group. The impaired processing of happy facial expressions is consistent with the previous finding that individuals with high ATs show weaker responses to gaze-triggered attentional shifts^111^ and facial mimicry^110^ during the processing of happy facial expressions than those with low ATs. The similar patterns of impaired detection of happy expressions among individuals with ASD and high ATs are in line with findings showing that ATs are continuously distributed in the general population extending into the clinically diagnosed ASD groups^105–108^. Extending the results of these previous studies, our results provide new evidence that the impairment of rapid detection of happy facial expression found in individuals with ASD is shared in individuals from the general population with high ATs.

The subjective emotional ratings in Experiment 1 showed less extreme valence and weaker arousal ratings for normal expressions in the ASD group than in the TD group. This result is in line with those of previous findings showing weak facial^91,92,94^ and autonomic^57,95–98^ emotional reactions to facial expressions. It is interesting to note that typical and atypical patterns for angry and happy expressions, respectively, shown in the RTs were not evident in the subjective emotional ratings in individuals with ASD and with high ATs. This suggests that the rapid detection and subjective emotional elicitation in response to emotional facial expressions may rely on partly dissociable psychological mechanisms in individuals with ASD and high ATs.

Although our results showing impaired rapid detection of happy, but not angry, facial expressions in individuals with ASD and high ATs were only partly consistent with our hypotheses based on previous findings in the ASD and ATs literature, the results fit with those reporting the effects of oxytocin on the rapid processing of emotional facial expressions. Oxytocin, a neuropeptide which acts as a neurotransmitter in distributed brain regions, has been shown to be reduced in individuals with ASD^114–117^ and its administration has been shown to improve their social cognition of faces^118,119^. Some previous studies tested the intranasal administration of oxytocin in TD individuals using the backward masking paradigm^120^, dot-probe paradigm^121^, and rapid serial visual presentation paradigm^122^ and found that oxytocin facilitated the rapid processing of happy facial expressions. One study showed that this effect was more evident in groups with high ATs^122^. By contrast, some studies reported that the oxytocin had an adverse effect on the rapid processing of negative facial expressions^123,124^. These data suggest that the present findings of impaired detection of happy facial expressions in individuals with ASD and high ATs may be related to their low levels of oxytocin. It may also be possible that the rapid detection of angry expressions depends on neural circuits that are not reliant on oxytocin and thus are preserved intact in autistic phenotypes.

What are the implications of results showing impaired rapid detection of happy facial expressions in individuals with ASD and high ATs? From the perspective of social psychology, happy facial expressions signal positive emotional states^125^ and affiliative intentions^126,127^. From the perspective of cognitive psychology, the detection of facial expressions is the first stage in conscious facial expression processing, which allows subsequent processes, such as the recognition of emotions and elicitation of interactive behaviors. The appropriate detection of facial expressions is critical, because human information-processing resources are limited and conscious processing is only available for stimuli from a central visual field^128^. Together, the psychological mechanisms for detecting happy facial expressions enable the efficient processing of important social stimuli from peripheral visual fields and promote interactive behaviors crucial to creating and maintaining affiliative relationships. Our results suggest that individuals with ASD and high ATs are impaired in this important initial processing stage. Several case reports have shown that individuals with ASD and high ATs have impaired affiliative relationships in real life (e.g., romantic partnerships and marriage)^129–132^. Our results suggest that these problems may be at least partially attributable to the impaired detection of happy expressions.

Several limitations of this study should be acknowledged. First, we tested adult participants only. The developmental trajectory of this phenomenon therefore remains untested. Previous studies have suggested the abnormal development of facial expression processing in individuals with ASD^56,63,104^. It may be that analogous atypical age-related changes appear in the impaired detection of happy expressions in individuals with ASD. Because even newborn infants show preference for happy facial expressions^133^, the impaired detection of happy facial expressions may be one of the primary problems in ASD. If so, promising directions for further research may include elucidating how this problem develops.

Second, our sample was small; hence, the results should be interpreted cautiously. Although we did not find impaired detection of angry facial expressions in the ASD group compared with the TD group, null group differences may be attributable to the lack of statistical power. In fact, several studies have found impairment in several processes, such as facial mimicry^91,93,94^ and autonomic arousal^95^ for angry expressions in ASD. Future studies with larger ASD samples may reveal the impaired detection of angry and happy facial expressions in individuals with ASD.

In summary, our experiments using the visual search paradigm with photographic stimuli for normal and anti-expressions of anger and happiness showed that RTs for normal vs. anti-expressions of happiness were shorter in the TD group but not in the ASD group. Similarly, detection of normal vs. anti-expressions of happiness was faster in the low ATs group but not in the high ATs group. These results suggest that the rapid detection of happy facial expressions is impaired in individuals with ASD and high ATs. We speculate that this impairment may possibly contribute to their difficulties in creating and maintaining affiliative social relationships.

## Methods

### Experiment 1

#### Participants

The ASD group consisted of 17 adults with ASD (5 females and 12 males; mean ± *SD* age = 26.9 ± 5.5 years). The diagnosis was made using the Diagnostic and Statistical Manual-IV-Text Revision^134^ via a stringent procedure in which every item of the ASD diagnostic criteria was investigated in interviews with participants and their parents (and professionals who helped them, if any) by at least two psychiatrists with expertise in developmental disorders. Only participants who met at least one of the four social impairment items (i.e., impairment in nonverbal communication including lack of joint attention, sharing interest, relationship with peers, and emotional and interpersonal mutuality) without satisfying any of the autistic disorder criteria, such as language delay, were included. Comprehensive interviews were administered to obtain information on the participants’ developmental histories for diagnostic purposes. Neurological and psychiatric issues other than those associated with ASD were ruled out. Participants were taking no medication. Full-scale intelligence quotients (IQs), measured by the Wechsler Adult Intelligence Scale, third edition (WAIS-III) (Nihon Bunka Kagakusha, Tokyo, Japan), of all participants in the ASD group fell within the normal range (mean ± *SD* = 112.8 ± 15.3; range, 86–134).

The TD group consisted of 17 adults (5 females and 12 males; mean ± *SD* age = 26.5 ± 4.8 years), who were recruited by personal contact. The TD participants were matched for age, *t*(32) = 0.02, *p* = 0.718, and sex, *χ* ^2^(1) = 0.00, *p* = 1.000, with the ASD group.

All participants were right-handed, as assessed by the Edinburgh Handedness Inventory^135^, and had normal or corrected-to-normal visual acuity. After the procedures were fully explained, all participants gave their informed consent to participate. This experiment was approved by the local ethics committee of the Primate Research Institute, Kyoto University, and was conducted in accordance with institutional ethical provisions and the Declaration of Helsinki.

#### Experimental design

The experiment was constructed as a three-factor mixed design, with group (TD, ASD) as a between-participant factor and stimulus type (normal, anti-expression) and emotion (anger, happiness) as within-participant factors.

#### Apparatus

The events were controlled by Presentation 14.9 (Neurobehavioral Systems, San Francisco, CA) implemented on a Windows computer (HP Z200 SFF, Hewlett-Packard Company, Tokyo, Japan). The stimuli were presented on a 19-inch CRT monitor (HM903D-A, Iiyama, Tokyo, Japan) with a refresh rate of 150 Hz and a resolution of 1024 × 768 pixels. The refresh rate was confirmed using a high-speed camera (EXILIM FH100, Casio, Tokyo, Japan) with a temporal resolution of 1000 frames/s. The responses were obtained using a response box (RB-530, Cedrus, San Pedro, CA), which offers 2–3 ms RT resolution.

#### Stimuli

The photographs of normal and anti-expressions of anger and happiness were used as target stimuli, and the photographs of neutral expressions were used as distractor stimuli. The schematic illustrations of the stimuli are presented in Fig. 1. The stimuli were identical to those used in previous studies^5,7–9^. Each individual face subtended a visual angle of 1.8° horizontally × 2.5° vertically.

Normal expressions were gray-scale photographs depicting angry, happy, and neutral expressions of a female (PF) and male (PE) model chosen from a facial expression database^136^. Neither model was familiar to any of the participants. No expression showed bared teeth.

Anti-expressions were created from these photographs using computer-morphing software (FUTON System, ATR, Soraku, Japan). First, the coordinates of 79 facial feature points were identified manually and realigned based on the coordinates of the bilateral irises. Next, the differences between the feature points of the emotional (angry and happy) and neutral facial expressions were calculated. Then the positions of the feature points for the anti-expressions were determined by moving each point by the same distance but in the opposite direction to that in the emotional faces. Minor color adjustments by a few pixels were performed using Photoshop 5.0 (Adobe, San Jose, CA). For a different use of “anti-expressions” see Skinner and Benton^6,137^.

Two types of adjustments were made to the stimuli using Photoshop 5.0 (Adobe, San Jose, CA). First, the photographs were cropped into a circle, slightly inside the frame of the face, to eliminate contours and hairstyles not relevant to the expression. Second, the photographs were prepared so that significant differences in contrast were eliminated, thereby removing possible identifying information.

We prepared eight positions, separated by 45 degrees and arranged in a circle (10.0° × 10.0°), for the presentation of stimulus faces. Stimuli occupied four of the eight positions; half were presented to the left and half were presented to the right side. A schematic illustration of one such stimulus display is presented in Fig. 1. Each combination of the four positions was presented an equal number of times. In the target-present trials, the position of the target stimulus was randomly chosen; however, the target stimulus was presented on the left side in half the trials and on the right side in the other half. In the target-absent trials, all four faces were identical and depicted neutral expressions.

#### Procedure

The experiment was conducted in an electrically shielded and soundproofed room (Science Cabin, Takahashi Kensetsu, Tokyo, Japan). Participants sat in chairs with their chins fixed into steady positions 80 cm from the monitor. They were asked to keep their gaze on the fixation cross (0.86° × 0.86°) at the center of the display when the cross was presented. Before the experiment began, participants engaged in 20 practice trials to gain familiarity with the apparatus.

The experiment consisted of a total of 432 trials presented in six blocks of 72 trials, with an equal number of target-present and target-absent trials. The trial order was pseudo-randomized. In each trial, the fixation cross was presented for 500 ms and then the stimulus array consisting of four faces was presented until participants responded. Participants were asked to respond as quickly and as accurately as possible by pushing the appropriate button on a response box using their left or right index finger to indicate whether all four faces were the same or one face was different. The position of the response buttons was counterbalanced across participants.

After the visual search task, participants engaged in the rating task for the target and distractor (neutral) facial stimuli. The stimuli were presented individually. They were asked to evaluate each stimulus in terms of emotional valence and arousal (i.e., the nature and intensity of the emotion, respectively, that participants felt when perceiving the stimulus expression)^112^ using a nine-point scale ranging from 1 (negative and low arousal, respectively) to 9 (positive and high arousal, respectively). The order of facial stimuli and rating items during the rating task were randomized and balanced across participants. Although we also asked participants to rate familiarity (i.e., the frequency with which they encountered facial expressions such as those depicted by the stimulus in daily life) and naturalness (i.e., the degree to which the expression depicted by the stimulus seemed natural) of stimuli as in previous studies^5,7–9^, these data are not reported here, because they were irrelevant to the purposes of the study.

#### Data analysis

All statistical tests were performed using SPSS 16.0 J software (SPSS Japan, Tokyo, Japan).

For RT analysis, the mean RTs of correct responses in target trials were calculated for each condition, excluding measurements ±3 *SD* from the mean as artifacts. To satisfy normality assumptions for the subsequent analyses, the data were subjected to a log transformation. The log-transformed RT was analyzed using a three-way repeated-measure ANOVA with group (TD, ASD) as a between-participant factor and stimulus type (normal, anti) and emotion (anger, happiness) as within-participant factors. Follow-up analyses of significant interactions for the simple effect were conducted^138^. When higher-order interactions were significant, the main effects or lower-order interactions were not subjected to interpretation because they would be qualified by higher-order interactions^139^.

Preliminary analyses for accuracy (mean% ± *SD*; normal anger: 96.4 ± 4.0, 94.1 ± 5.9; normal happiness: 97.1 ± 2.4, 95.6 ± 4.2; anti-anger: 85.6 ± 11.3, 81.9 ± 13.0; and anti-happiness: 84.6 ± 12.2, 82.7 ± 13.3 for TD and ASD groups, respectively) showed no evidence of a speed–accuracy trade-off (i.e., lower accuracy for faster responses) and group difference. Hence, we report only the RT results, as in previous studies^5,8,9^.

Each rating of valence and arousal was analyzed using an ANOVA and follow-up analyses identical to those used for the RT analysis. In addition, to compare the rating scores of normal/anti-expressions of anger and happiness with those of neutral expression, multiple comparisons with the Bonferroni correction were conducted; the *α* level was divided by the number of tests performed (i.e., 4 per group).

The *α* level for all analyses was set to 0.05.

### Experiment 2

#### Participants

A total of 51 adults (26 females and 25 males; mean ± *SD* age, 22.5 ± 4.5 years), who were recruited by advertisement, were initially investigated. All of these participants completed the Japanese version of the AQ, which includes a 50-item self-rating questionnaire to measure ATs^105^ and visual search experiments. Nine participants showed high rates of error in trials (>50% under at least one of normal anger, normal happiness, anti-anger, or anti-happiness conditions); their data were not included in the analysis. The remaining participants were divided based on a median split for the AQ scores into two groups. The median AQ score (21) was in good agreement with that of a previous standardization study with young Japanese participants (mean ± *SD*, 20.7 ± 6.4)^140^. The 18 participants (10 females and 8 males; mean ± *SD* age, 23.3 ± 3.3 years) whose AQ scores were below 21 points (mean ± *SD = *17.2 ± 2.7) were classified as the low ATs group. The 20 participants (9 females and 11 males; mean ± *SD* age, 21.7 ± 3.6 years) whose AQ scores were above 21 points (mean ± *SD* = 27.4 ± 5.0) were classified as the high ATs group. Four participants had scores equal to the median of all participants’ scores; their data were excluded from the analysis. All participants were right-handed, as assessed by the Edinburgh Handedness Inventory^135^, and had normal or corrected-to-normal visual acuity. A psychiatrist or psychologist administered a short structured diagnostic interview using the Mini-International Neuropsychiatric Interview^141^; no neuropsychiatric problems at a clinical level were detected in any of the participants. Full-scale IQs were also measured by the WAIS-III (Nihon Bunka Kagakusha, Tokyo, Japan) and all participants showed scores in a normal range (mean ± *SD* = 122.2 ± 7.2 and 122.6 ± 9.6, in the low and high ATs groups, respectively). A significant group difference between the low and high ATs groups was found in the AQ scores, *t*(36) = 7.68, *p* = 0.000, but not terms of age and full-scale IQs, *t*(36) = 0.56 and 0.14, *p* = 0.576 and 0.891, respectively, and sex, *χ* ^2^(1) = 0.42, *p* = 0.516. After the procedures were fully explained, all participants provided informed consent for participation. This experiment was part of a larger project investigating personalities and mental health. The experiment was approved by the local ethics committee of the Primate Research Institute, Kyoto University, and conducted in accord with institutional ethical provisions and the Declaration of Helsinki.

#### Experimental design

The design of Experiment 2 was identical to that used in Experiment 1 with one exception: the group factor included the levels of low vs. high ATs.

#### Apparatus, stimuli, and procedure

The apparatus, stimuli, and procedure were identical to those used in Experiment 1.

#### Data analysis

The method of data analyses was almost identical to that used in Experiment 1, with one exception: the group factor included the levels of low vs. high ATs. Preliminary analyses for accuracy (mean% ± *SD*; normal anger: 92.0 ± 10.4, 93.3 ± 5.3; normal happiness: 84.2 ± 14.2, 83.0 ± 11.1; anti-anger: 84.5 ± 12.4, 86.7 ± 10.2; anti-happiness: 96.2 ± 8.9, 98.1 ± 1.9 for low and high ATs groups, respectively) showed no evidence of a speed–accuracy trade off or of a group difference; hence, we report only the RT results.
